# Common international trends in football stadium attendance

**DOI:** 10.1371/journal.pone.0247761

**Published:** 2021-03-03

**Authors:** Jan C. van Ours

**Affiliations:** 1 Erasmus School of Economics, Erasmus University Rotterdam, Erasmus Center of Applied Sports Economics (ECASE) and Tinbergen Institute (Rotterdam), Rotterdam, The Netherlands; 2 Department of Economics, University of Melbourne, Parkville, Australia; 3 CEPR, London, United Kingdom; Ghent University, BELGIUM

## Abstract

This paper examines long-term developments in stadium attendance in professional football in the Netherlands. As in many other European countries attendance had a U-shaped development with the lowest numbers in the mid-1980s. The developments in the Netherlands do not seem to have been affected by hooliganism but by socioeconomic factors. Furthermore, the association with stadium attendance in other European leagues in particular the English Premier League is very high. This suggests that stadium attendance is affected not only by national developments but also by common international trends in the interest in football matches.

## 1 Introduction

For many supporters, football is like a religion. During the season, they usually meet once a week alternating between the home stadium and an away stadium. Songs are sung, joy and happiness are shared in case of a win, sadness and disappointment are internalized in the companionship of fellow supporters. For many supporters, being at a match of their favorite team is like sitting in a roller-coaster of emotions. The days after a loss are depressing, the days after a win happiness is boasted, they are full of satisfaction about their favorite team, football as a sport and sometimes about life in general. For some supporters, visiting a football stadium is an integral part of their life. Nevertheless, not every football supporter will visit a stadium whatever the costs may be, financially or emotionally. Buying a seasonal ticket or a day ticket to attend a match is like consuming a service offered by a football club. Therefore, stadium attendance is subject to the usual determinants of consumer demand but is also influenced by some sport-specific characteristics.

While there is similarity between consumer demand and demand for a stadium seat there are clear differences at the supply side between incentives of regular firms and those of sports clubs. Whereas regular firms may aim for a monopolistic market position to maximum profits for the sports club a monopoly would destroy the business (Neale [1]). Sports fans are looking for excitement not for boring games where the outcome can be guessed long in advance. Szymanski [2] for example concludes from a comparison of attendances of Premier League matches and FA Cup matches—in which over time the difference in strength between the two competing teams increased a lot—that a drop in competitive balance reduced stadium attendance.

Borland and MacDonald [3] provide an extended overview of the determinants of the demand for sports in general and stadium attendance in particular. They mention five main categories of determinants of stadium attendance: Consumer preferences (supporting a club), economic determinants (price, travel costs, income, market size, availability of substitutes, macroeconomic factors), quality of viewing (quality of seating, timing of the contest), characteristics of the sporting contest (uncertainty of outcome, success of competing teams, quality and significance of the match) and stadium capacity. Some of these determinants relate to stadium attendance of single matches, other determinants are important for seasonal attendance since a lot of attendants are supporters who have seasonal tickets. As to the uncertainty of outcome, there is match uncertainty depending on the strength of the two teams playing and seasonal uncertainty related to end-of-season matches that may determine who wins the league, who is relegated, and so on.

Recent empirical studies on stadium attendance are usually based on match-level data from a limited number of seasons in a single country investigating the relationship with among others the uncertainty of outcome. Besters et al. [4] for example analyze 18 seasons of teams from the top football league in the Netherlands finding that the attendance of individual matches in Dutch professional football is related to loss aversion more than to preference for uncertain outcomes. Furthermore, team quality is important while towards the end of the season, outcome uncertainty with respect to the final ranking becomes important. Other examples of short-run studies are García and Rodríguez [5] who investigate the determinants of stadium attendance at the match level in the Spanish top league over four seasons, Cox [6] who studies eight seasons of English Premier League football, Martins and Cró [7] who study four seasons of Portuguese first division football, Di Domizio and Caruso [8] studying five seasons of Italian football. Reade [9] is somewhat of an outlier as he analyzes for a period of 130 years about 200,000 match-level observations from English football focusing on the relationship between competitive balance and stadium attendance. Apart from determinants related to stadium visits themselves, there is interaction between stadium attendance and viewing matches on television. Buraimo [10] for example concludes that there is a positive relationship between the two in the sense that crowded stadiums are more attractive to watch on television. Similarly, one can imagine that watching a match on television may stimulate to desire to be present in the stadium.

Whereas there are quite a few studies using short-run match-level data, there are not many economic studies that have a long-term perspective on stadium attendance and thus are able to consider the determinants of long-run developments. Dobson and Goddard [11, 12] are among the exceptions studying a period of almost 70 (and 50) years of English football. As is common in these long-term studies seasonal averages of club-level match attendance are used as dependent variables. Dobson and Goddard find that ticket-prices have a significant but small effect, while success of a club is a major determinant of stadium attendance. A peculiarity of stadium attendance in English football is the dip in the 1980s which Dobson and Goddard [11] attribute to the economic recession and hooliganism that had its heyday in England. The recovery in attendance started late 1980s when technological developments made television-broadcasting possible through cable and internet thus attracting attention to the excitement and joy of attending a live football match (Koning [13]). The interaction between attending a match and watching a match on television is a recent phenomenon i.e. happening in the past decades. It cannot explain the big drop in stadium attendance in the period early 1970s to late 1980s. Whereas for some time broadcasting a match on television was thought to be at the expense of stadium attendance in recent decades there seems to be complementarity rather than substitution. In that sense, the increase in football watching on television may have stimulated stadium attendance.

The current paper has a focus on long-run developments presenting an analysis of seasonal stadium attendance in Dutch professional football from the start in 1956/57 to 2018/19, the last full season before the Covid-19 crisis forced stadiums to remain empty. The set-up of this paper is as follows. Section 2 provides an international perspective of long term developments in stadium attendance comparing six football leagues indicating that in the past 60 seasons cross-country correlation in attendance has been remarkably high. The dip in stadium attendance in the 1980s was a phenomenon that was present in the top tiers of professional football of quite of few countries. Furthermore, this section describes football stadium attendance in the Netherlands in the past 63 seasons in more detail. Section 3 discusses potential determinants of seasonal stadium attendance distinguishing between club-specific time-varying determinants and season-specific club-invariant determinants. Club-specific determinants are seasonal performance of the club and stadium capacity. Season-specific determinants that may affect clubs across the board are the socioeconomic situation, hooliganism, recreational developments and interest in football. These determinants are represented by unemployment rate, arrests because of football hooliganism, cinema visits as an indicator for recreational developments and stadium attendance in the English Premier League representing the international trend in interest in high-quality football matches. Section 4 presents the empirical analysis of long-run developments in football stadium attendance in the Netherlands. This is done in two stages. In the first stage, club-specific stadium attendance is specified in a linear regression with fixed effects for clubs and seasons, seasonal performance indicators and stadium capacity as explanatory variables. In the second stage, the seasonal fixed effects from the first stage are related to unemployment rate, stadium attendance in the Premier League, cinema visits and hooliganism. Section 4 also presents an exploratory analysis of stadium attendance in the top leagues of England, Germany, Belgium and France. In the exploratory analysis developments in seasonal averages of stadium attendance are related to unemployment, a time trend and international spillovers from stadium attendance in other countries. Section 5 concludes that stadium attendance is influenced by club-specific as well as season-specific factors. Over time, unemployment rates have been important for stadium attendance in the Netherlands whereas hooliganism was not. There is also a strong association between football stadium attendance in the Netherlands and England suggesting that there are common international trends in the interest in football. A simple model with unemployment rates, a time trend and average football stadium attendance in other countries not only works well for the Netherlands but also for England and to some extent in Belgium and Germany while it does not work so well for football stadium attendance in France.

## 2 Developments in stadium attendance

### 2.1 International developments

**Fig 1:**
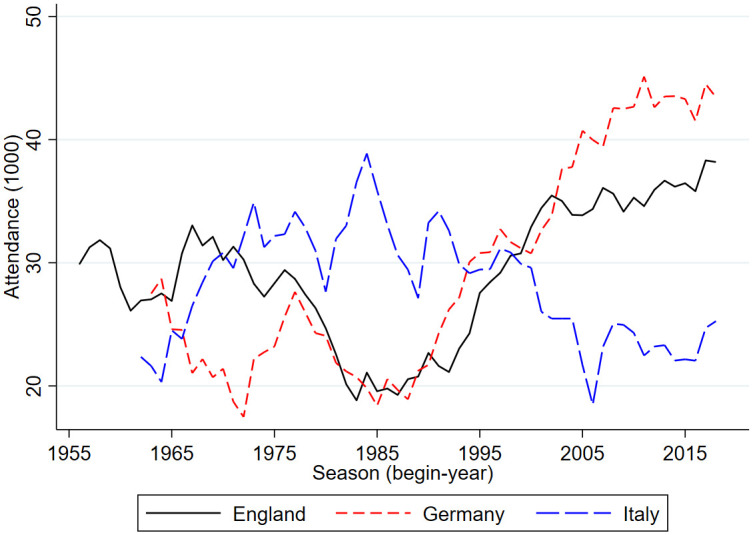
Stadium attendance professional football; England, Germany, Italy; 1956/57-2018/19 (1000).

**Fig 2:**
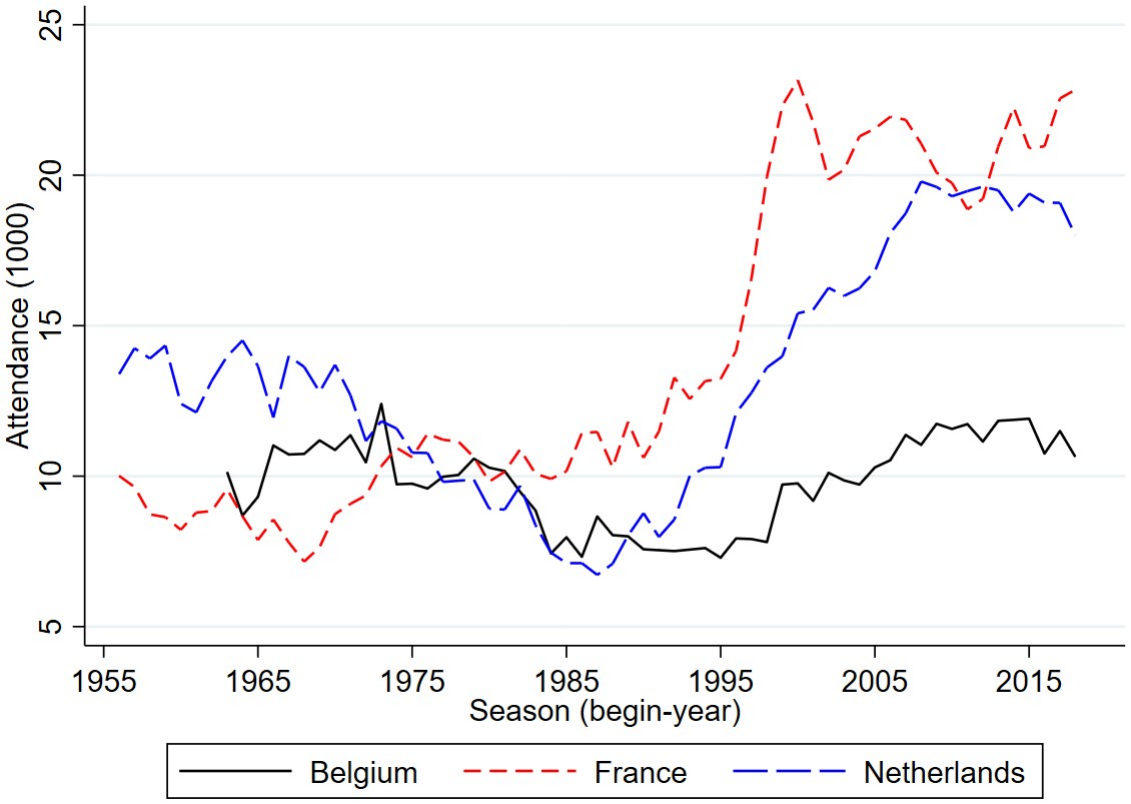
Stadium attendance professional football; Belgium, France, The Netherlands; 1956/57-2018/19 (1000).

Figs 1 and 2 provides an graphical overview of international developments in football stadium attendance. Fig 1 shows developments in the top leagues of England, Germany and Italy. Fig 2 shows developments in the top leagues of Belgium, France and the Netherlands. Clearly, since the mid-2010s the German Bundesliga had the highest number of stadium attendants. Over the past years this was over 43,000 per match. Over the past years, the English Premier League had about 37,000 attendants per match, the Spanish La Liga had about 27,000 attendants, the Italian Serie A about 23,000, the French Ligue 1 about 22,000 and the Dutch Eredivisie had about 19,000 attendants per match. The Belgium Pro League is by far the smallest league with in recent years about 11,000 match attendants.

From 1960 onward, over time in most but not all countries the number of match attendants went up after a dip in the 1980s. Also, for many but not all countries the average number of match attendants in the early periods is lower than in the last periods. Belgium and Italy are two countries where the numbers at the end are very similar to those in the beginning. In the second half of the 1960s, in Belgium there were on average 10,000 match attendants, in the late 2010s this was a little over 11,000. In Italy, these numbers were 24,700 and 23,200. The difference between the two countries is that in Belgium there is a dip in the early 1990s with less than 8,000 match attendants while in Italy there is a peak in the second half of the 1980s of almost 34,000. Clearly, the development of stadium attendance in Italy since the mid 1980s is very different from developments in the other five countries. Whereas in the other five countries there is a clear and substantial increase, in Italy there is a downward trend. The peak in attendance in the 1980s in Italy is perhaps related to Italy being one of the top leagues in Europe with a lot of international stars and Italy winning the World Cup in 1982. According to Di Domizio and Caruso [8] the developments in Italy are also related to hooliganism and match-fixing scandals. Hooligans in Italy were well-organized and often violent such that major fights surrounding a match caused later matches to be played behind closed doors thus lowering average stadium attendance.

Table 1 shows pairwise correlations in stadium attendance based on annual data. The correlation is very high between the Netherlands and England (0.93) but also the correlations between England, France and Germany are high. Italy is a clear outlier. There is even a negative correlation between the number of annual match attendants in Italy and all other countries. The correlation in attendances in most leagues except for Italy could be caused by common determinants such as socioeconomic developments, developments in hooliganism, interactions between being present in the stadium or watching a match on television. Alternatively, there could be international spillover effects in interest to watch a football match in the stadium. If socioeconomic developments are important, economic cycles may also have induced correlation in stadium attendance. In particular unemployment rates in Western Europe are highly correlated. Like Dobson and Goddard [11], Szymanski and Drut [14] suggest that the drop in stadium attendance in England in the 1980s is related to the world-wide economic recession and hooliganism. Possible cross-border copycat behavior among football hooligans is potentially another reason why developments over time are similar in the five countries. In the empirical analysis, the relationship between attendance and hooliganism will be addressed.

**Table 1:** Pairwise correlations annual seasonal attendances.

	Belgium	England	France	Germany	Netherlands
England	0.72 ***				
France	0.30 **	0.68 ***			
Germany	0.46 ***	0.78 ***	0.88 ***		
Netherlands	0.70 ***	0.93 ***	0.71 ***	0.88 ***	
Italy	-0.50 ***	-0.68 ***	-0.51 ***	-0.72 ***	-0.79 ***

Note:

*** p<0.01,

** p<0.05

Source: www.european-football-statistics.co.uk/attn.htm

### 2.2 The Netherlands

The top tier in Dutch football is called “Eredivisie” (meaning “Honorary division”). The second tier of professional football in the Netherlands is called “First division”. For some time, there was also a third tier called “Second division”. As shown in Fig 2, up to the early 1970s average match attendance in the Eredivisie fluctuated around 12,500. Then the numbers started to decline to reach the lowest level of less than 7,000 in the season 1987/88. From the early 1990s, there is a steady increase up to almost 20,000 in 2008/09 to decline somewhat in later years.

Over the years, in the Netherlands quite a few professional football clubs ceased to be while other clubs merged into a new club with a different name. Sometimes a merger of two clubs got the name of one of the clubs. In other situations clubs changed their name to emphasize the name of the city of residence or clubs introduced small changes in their name. One of the examples is the name change from Feijenoord to Feyenoord. The pronunciation of the name in Dutch did not change because of that but from an international point of view the pronunciation became much easier after the name change. The S1 Appendix provides a detailed overview of mergers between clubs and name changes whereby in this paper the most recent name is used. To create a balanced panel two criteria were used: (1) the club played professional football in all 63 seasons and (2) the club played at least one season in the Eredivisie. In total 30 clubs fulfilled both criteria. Some summary statistics of the clubs are shown in Table 2. There are four clubs that were present in the Eredivisie all the time: Ajax, FC Utrecht, Feyenoord and PSV. Helmond Sport was present in the top tier for only two seasons, FC Eindhoven for three seasons. About half of the clubs spent at least one season in the second division. Average seasonal stadium attendance per match ranges from the top end with Feyenoord (34,000), Ajax (29,000) and PSV (23,000) to the low end with FC Dordrecht and Helmond Sport who attracted less than 3,000 attendants per match.

**Table 2:** Summary statistics by club; 1956/57—2018/19.

	Club	Number of Seasons			Attendance (1000)	Capacity (1000)
		Ere divisie	First division	Second division		
1	ADO Den Haag	45	18	0	9.0	15.7
2	Ajax	63	0	0	28.6	46.8
3	AZ Alkmaar	42	19	2	8.9	13.6
4	De Graafschap	21	34	8	6.5	9.5
5	FC Den Bosch	15	45	3	4.4	9.3
6	FC Dordrecht	6	53	4	2.7	5.4
7	FC Eindhoven	3	58	2	3.3	6.1
8	FC Groningen	52	11	0	12.6	17.4
9	FC Twente	61	2	0	13.1	19.8
10	FC Utrecht	63	0	0	12.3	20.2
11	FC Volendam	25	38	0	4.5	9.2
12	Feyenoord	63	0	0	34.0	53.3
13	Fortuna Sittard	32	31	0	5.9	12.9
14	Go Ahead Eagles	31	29	3	6.7	12.0
15	Helmond Sport	2	54	7	2.8	5.2
16	Heracles Almelo	19	42	2	5.3	8.3
17	MVV Maastricht	36	27	0	6.2	12.5
18	NAC Breda	50	13	0	10.5	15.4
19	NEC Nijmegen	40	15	8	8.2	15.5
20	PEC Zwolle	19	29	15	5.2	8.5
21	PSV	63	0	0	23.0	27.4
22	Roda JC Kerkrade	50	5	8	8.7	15.1
23	SBV Excelsior	22	37	4	3.2	7.0
24	SBV Vitesse	34	25	4	10.5	15.8
25	SC Cambuur	7	52	4	5.6	9.3
26	sc Heerenveen	27	24	12	10.3	13.4
27	Sparta Rotterdam	53	10	0	8.9	17.9
28	Telstar	14	48	1	3.4	7.5
29	VVV-Venlo	22	37	4	5.1	9.0
30	Willem II	43	20	0	8.4	13.4
	Average	34	26	3	9.3	15.1

Note: The second division was terminated after season 1970/71. Seasonal stadium capacity for a club is proxied by the match with the highest attendance in that season.

## 3 Determinants seasonal stadium attendance

The developments in stadium attendance are partly club-specific and partly driven by general developments, i.e. factors that vary over time but influence all the clubs in a similar way. Table 3 shows descriptives of the variables used in the analysis. The S1 Appendix provides definitions and sources for the variables.

**Table 3:** Data descriptives; 1956/57—2018/19.

Variable	Mean	Minimum	Maximum	Observations
a. Club-specific				
Attendance	9,266	661	52,987	1890
Capacity	15,075	1200	68,000	1890
First division	0.41	0	1	1890
Second division	0.05	0	1	1890
Points/100	0.49	0.13	1.01	1890
Ranking/10	0.89	0.1	2.1	1890
Goal difference/100	0.03	-0.73	0.90	1890
b. Season/year variables				
Unemployment rate	4.8	0.8	10.7	63
Premier League (1000)	29.2	18.8	38.3	63
Hooliganism related arrests	1326	652	2401	27
Cinema visits (mln)	27.9	13.7	69.1	63

### 3.1 Club-specific determinants

Over the past decades many clubs have changed the capacity of their stadium, most often by expanding it but sometimes by reducing the capacity for example when standing positions were abolished and attendants had to take a seat. Sometimes clubs renovated their stadium while other clubs built a new stadium.

Between seasons, stadium capacity may not be exogenous to stadium attendance. If a club is very popular in terms of people attending the stadium the club may decide to expand its stadium. Furthermore, stadium capacity is not a fixed number. Especially in the early years of professional football in the Netherlands capacity could easily be expanded by introducing additional space sometimes as additional places to stand. For example, until December 2005 the Oosterpark stadium—home to FC Groningen—had a formal capacity of 12,500 seats but the stadium could be expanded to 20,000 by adding standing places. Clubs could also change stadium if they expected a large crowd. Ajax for example played until season 1995/96 in De Meer with a capacity of 29,500. However, some of their matches were played in the Amsterdam Olympic Stadium which had a capacity of 42,000. To deal with this flexibility issue stadium capacity in a particular season is defined as the highest number of attendants of a single match in that season. As shown in panel a of Table 3 there are 1890 observations (30 clubs—63 seasons) in which attendance ranged from 661 to 52,987 and stadium capacity from 1200 to 68,000. Note that the stadium capacity is determined by the highest attendance during a particular match of a club while the highest attendance reported in Table 3 is the highest attendance averaged over a season for a club. Of the observations 41% is from the second tier—the first division—and 5% from the third tier—the second division.

Stadium attendance for a club in a particular season may also be affected by the success of a club in that season. There are various indicators to measure success such as the number of points achieved, the final position in the league table, the goal difference or achieving a championship. Whereas an increase in average stadium capacity may lead to an increase in average stadium attendance this is different for success as the success of one club is always at the expense of other clubs. Table 3 shows that the average number of points at the end of the season ranged from 13 to 101 with an overall average of 49. These points are calculated on the basis of the current system where a club gets three points for a win, one point for a draw and zero points for a loss. In reality, until season 1994/95 clubs got two points for a win in stead of three. End-of-season ranking ranges from 1 to 21 with average of 8.9. The end-of-season goal difference ranges from -73 to +90 with an average of +3.

### 3.2 Season-specific determinants

**Fig 3:**
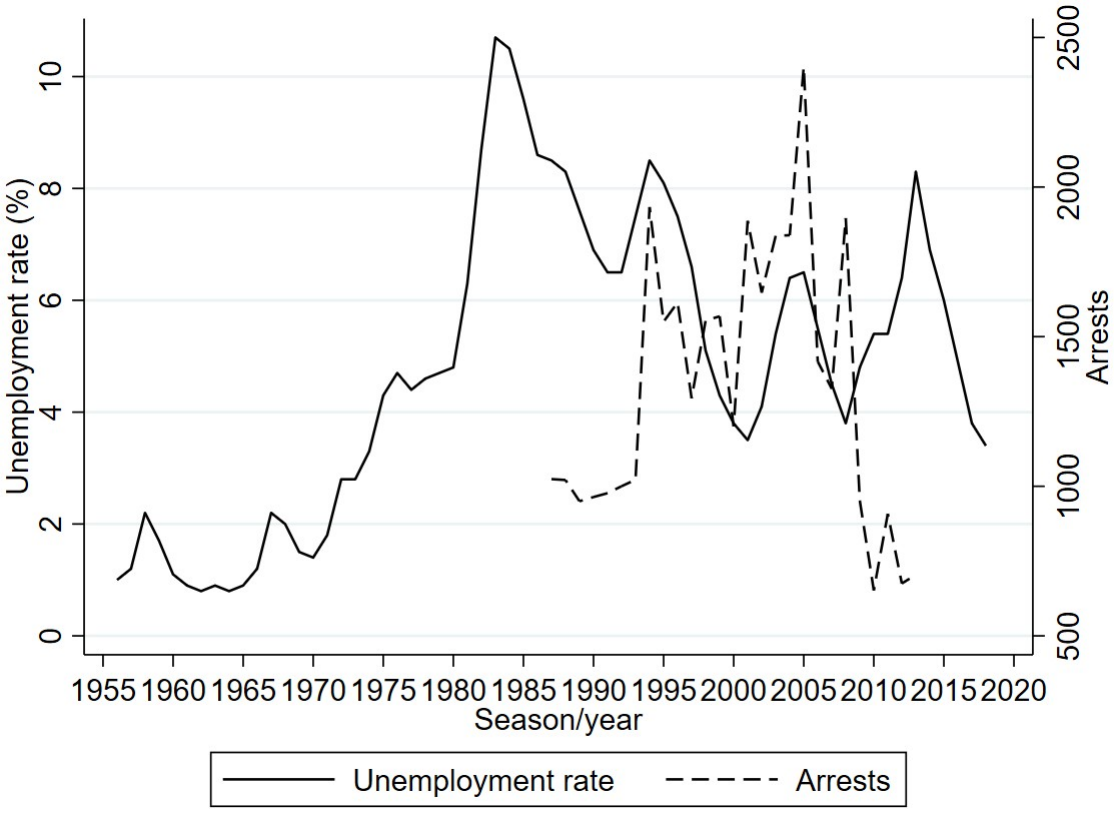
Unemployment rate (%) and arrests; 1956-2018.

**Fig 4:**
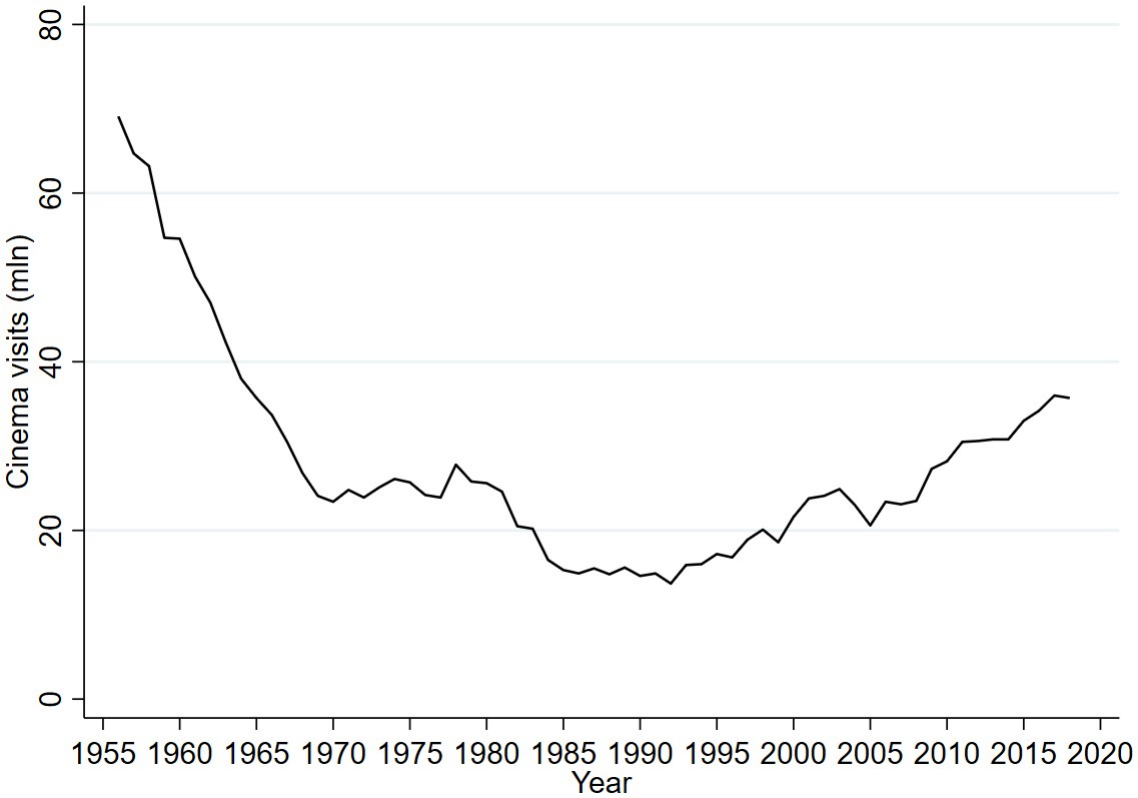
Cinema visits; 1956-2018.

Stadium attendance is likely to be influenced by socioeconomic developments such as the unemployment rate for which an overview is provided in panel a of Fig 3. Clearly, from the early 1970s onward, the unemployment rate increased substantially from less than two percent to more than 10 percent in the middle of the 1980s. After that, unemployment rates went down but with substantial annual fluctuations. Previous studies suggest that hooliganism affected stadium attendance in for example England and Italy. Whether this is also the case in the Netherlands is not clear. Systematically collecting information about football hooliganism in the Netherlands started in 1987. Linckens and Berghuis [15] analyzed information about football hooligan arrests in 1987 concluding that about 80 percent of the arrests was for violations outside the stadium, 40 percent of the hooligans were minor, i.e. younger than 18 years while about half of the hooligans had been in contact with justice on a previous occasion for issues unrelated to football hooliganism. Spaaij [16] argues that before the 1970s there was little football hooliganism in the Netherlands. In the 1980s and 1990s not much changed in terms of the quantity of the hooliganism. The event that is considered to be the starting point of football hooliganism in the Netherlands is the May 1974 UEFA Cup final match between home team Feyenoord and the London team Tottenham Hotspur. Visiting supporters attacked the home supporters and more than 200 people were injured. The first domestic stadium riot that was televised was in October 1976 and concerned supporters of FC Utrecht and Ajax. November 1983, there was life broadcasting of within-stadium fighting between supporters of (again) Feyenoord and Tottenham Hotspur. Over the course of time, various measures were introduced to prevent hooliganism in and around football stadiums: home and away supporters were physically separated, large numbers of police were present at matches that were anticipated to be risky, stadiums were transferred into all-seats, i.e. all supporters were supposed to be seated, CCTV (Closed circuit television) was installed, etcetera. This reduced football hooliganism within and outside stadiums a lot but the hooliganism was shifted to other places such as city centers and train stations. Overall football hooliganism was not reduced. Schaap et al. [17] analyze match level data over the period 2006 to 2011 on hooliganism in the Netherlands in an attempt to evaluate policy measures aiming to reduce football hooliganism. They find that matches played early in the day and in daylight have a smaller probability of hooliganism occurring. They also find that alcohol prohibition within the stadium increases the probability of an incident outside the stadium attributing this to a “waterbed effect”. Fig 4 confirms that the number of arrests fluctuated a lot with no clear upward or downward pattern.

Another potential determinant of stadium attendance is the recreation of the Dutch population. As an indicator for recreational activities in relation to stadium attendance visits to the cinema are used. After all, both represent behavior of people consuming a service outside their home, i.e. a movie or a football match. The idea is not that cinema visits had a causal effect on football stadium attendance. Rather cinema visits picked up a trend in outgoing behavior that may have affected stadium attendance as well. Fig 3 shows a strong decline in cinema visits probably related to the introduction and later expansion of television broadcasting in the Netherlands. From the mid-1980s onward cinema visits increased possibly reflecting a change in outgoing behavior. To examine possible international spillovers affecting stadium attendance in the Netherlands, the attendance in the English Premier League is added as right-hand side variable. As indicated in the table at the bottom of Fig 1 at the level of the top leagues correlation with England is the strongest.

Panel b of Table 3 provides the descriptives for the seasonal data. Over the 63 years the unemployment rate ranges between 0.8 and 10.7 percent of the labor force with an average of 4.8%. Premier League attendance ranged from 18,800 to 38,300 with an average of 29,200. Cinema visits ranged from 13.7 million to 69.1 million with an average annual number of 27.9. The information about hooliganism is limited to 27 seasons in which the number of arrests ranged from 652 to 2401.

## 4 Empirical analysis

### 4.1 Set-up of the analysis

The data on stadium attendance is multi-level, i.e. there is information of 30 clubs over a period of 63 seasons. The observations at the club level are nested within the higher level of seasons so there is a natural hierarchy in the data. Within and between seasons stadium attendance may be influenced by club-specific factors such as stadium capacity, the league (eredivisie, first division, second division) and the performance of the club. Additionally, between seasons stadium attendance may be influenced by socioeconomic developments, hooliganism, trend-like changes, changes in recreational preferences, and international spillovers. Some determinants of stadium attendance are club-specific, others are common to all clubs but season-specific. To identify the relationship with stadium attendance, for the first type of determinants 1890 data-points are available. For the second type of determinants 63 data-points are available. To account for these differences, the estimations are performed in two stages. In the first stage, a least squares dummy variables regression is done. The dependent variable *y*_*it*_ is the log of stadium attendance of club *i* in season *t*:

$$\begin{array}{c} y_{it}=\alpha_{i}+\beta x_{it}+\gamma_{t}D_{t}+\varepsilon_{it} \end{array}$$ (1)

where *x*_*it*_ is a vector of club-specific time varying explanatory variables. These variables include performance indicators, stadium capacity and two dummy variables indicating in which division the club played during season *t*. Furthermore, *D*_*t*_ are seasonal dummy variables, and *α*_*i*_ represents club fixed effects. There is no information available about ticket prices. To the extent that these are club-specific they are absorbed in the club fixed effects. To the extent that they have a trend-like development they are absorbed in the time trend which is included in the second stage. Also, ticket prices are only part of the costs of visiting a match. Other costs involved are travel costs and costs of leisure time. Furthermore, *β* is a vector of parameters, *γ*_*t*_ a vector of seasonal fixed effects and *ε*_*it*_ is an error term.

There are several issues related to the first stage of the analysis. Wallrafen et al. [18] argue that competition between the top leagues and lower divisions may have increased over time because of increasing overlap in the scheduling of matches. Based on an analysis of German fourth division match data they conclude that this is indeed the case. The assumption underlying the use of a first division dummy (and a second division dummy) is that such increased competition has not occurred but has remain constant over time. Furthermore, as discussed earlier stadium capacity may not be exogenous to stadium attendance as the presence of a large number of potential attendants may induce clubs to expand their stadium. Nevertheless, stadium capacity does not change within a season and it provides an upper limit to stadium attendance. The final issue is whether performance is exogenous to average stadium crowds which both are end-of-season variables. It is possible that big stadium crowds have a positive effect on match performance. Nevertheless, it is difficult to imagine that the size of the crowd has an effect on seasonal performance. After all, only half of the matches during a season are played in the home stadium. And, for many clubs seasonal ticket holders are an important part of the stadium attendants. The fluctuating part of the stadium attendants is more likely to be attracted by good performance rather than the other way around.

In the second stage of the analysis, to explain developments over seasons a model is estimated using ${\hat{\gamma }}_{t}$ as the dependent variable. The regression model in the second stage is given by

$$\begin{array}{c} {\hat{\gamma }}_{t}=\delta z_{t}+u_{t}, \end{array}$$ (2)

where *z*_*t*_ is a vector of time-varying explanatory variables. When a variable is defined by calendar year they refer to the first calendar year in a season, i.e. if the season is 1980/81, the calendar year is 1980. Furthermore, *δ* is a vector of parameters and *u*_*t*_ is an error term. The parameter estimates in the second step are unbiased and since the number of observations is quite large (63), the standard errors are estimated accurately (Bryan and Jenkins [19]).

### 4.2 Parameter estimates

Table 4 shows the parameter estimates for the first stage of the analysis where the log of attendance per club per season is related to the division, performance indicators and log stadium capacity. Compared to the Eredivisie, attendance is lower in the first and second division. Of the three performance indicators the number of points in a season is insignificantly different from zero, while ranking and goal difference are both highly significant. It is no surprise that it is not possible to estimate the separate contribution of each of the three performance indicators. Naturally, a high goal difference will generate more points and more points cause a higher rank. The pairwise correlations are high: between points and goal difference 0.94; between rank and points and rank and goal difference 0.90. As a win for individual matches always implies three points but the three points can be obtained by a variety of goal differences, goal difference is perhaps a more refined indicator for entertainment than points. Stadium capacity has a significant positive effect on stadium attendance.

**Table 4:** Parameter estimates stadium attendance; first stage—club-specific effects.

	1956/57-2018/19		1987/88-2013/14	
	(1)	(2)	(3)	(4)
First division	-0.31***	-0.31***	-0.44***	-0.44***
	(0.03)	(0.03)	(0.05)	(0.05)
Second division	-0.42***	-0.42***		
	(0.07)	(0.07)		
Ranking/100	-0.80**	-0.65***	-0.63	-0.64**
	(0.37)	(0.23)	(0.44)	(0.30)
Goal difference/100	0.29***	0.24***	0.17**	0.18***
	(0.05)	(0.05)	(0.07)	(0.06)
Points/100	-0.13		0.00	
	(0.16)		(0.18)	
Log Stadium Capacity	0.73***	0.73***	0.57***	0.57***
	(0.03)	(0.03)	(0.05)	(0.05)
Observations	1,890	1,890	810	810
R-squared	0.866	0.866	0.869	0.869

Note: Fixed effects for seasons and clubs are included; R-squared is within. Robust standard errors in parentheses;

*** p<0.01,

** p<0.05

In the second column seasonal points are removed as explanatory variable. This affects the parameter estimate for ranking somewhat, but leaves the other parameter estimates largely unaffected. Columns (3) and (4) show equivalent estimates over the time period 1987/88 to 2013/14, the time period over which there is information about hooliganism. During this time period the second division no longer existed. Although the parameter estimates are slightly different from those in the first two columns the magnitudes of the effects are very much the same.

From column (2) of Table 4 it follows that compared to playing in the Eredivisie, in the first division stadium attendance was about 25% lower while playing in the second division reduced the number of attendants with another 10%-points. One place up in the final ranking on average generated close to 1% additional stadium attendance while 1 goal extra generated about 0.2% extra attendance.

Table 5 shows the parameters of the second stage regression. The dependent variables are the series of seasonal fixed effects from columns (2) and (4) of Table 4. Column (1) shows that the calendar time developments in stadium attendance are subject to a linear trend, which may represent developments in preferences but could also reflect population growth. Furthermore, the unemployment rate has a negative effect on stadium attendance. Column (2) shows that in addition to this the development of Premier League attendance has a significant positive parameter estimate. Column (3) shows that cinema attendance is positively related to stadium attendance. Columns (4) to (6) show what happens if the log of the number of arrests is included as additional variable. Except for cinema visits, the other parameters are not very much affected. The effect of arrests themselves is positive which is probably due to reverse causality, i.e. more arrests are possible with bigger crowds. Clearly, hooliganism did not have a negative effect on stadium attendance. This also implies that cross-country correlation of hooliganism is not a possible explanation for cross-country correlation in stadium attendance. As over the shorter period of time from 1987/88 to 2013/14 the effect on cinema visits disappears, also the cinema visits can be ignored as an indicator for change in leisure behavior affecting stadium attendance.

**Table 5:** Parameter estimates stadium attendance; second stage—seasonal effects.

	1956/57-2018/19			1987/88-2013/14		
	(1)	(2)	(3)	(4)	(5)	(6)
Log Urate	-0.26***	-0.12***	-0.08***	-0.15***	-0.10***	-0.10***
	(0.02)	(0.02)	(0.02)	(0.03)	(0.03)	(0.03)
Time/10	0.14***	0.08***	0.09***	0.27***	0.19***	0.19***
	(0.01)	(0.01)	(0.01)	(0.01)	(0.02)	(0.03)
Log Premier League		0.56***	0.41***		0.35***	0.35***
		(0.07)	(0.06)		(0.09)	(0.10)
Log Cinema Visits			0.16***			0.00
			(0.03)			(0.07)
Log Arrests				0.08***	0.03*	0.03
				(0.02)	(0.02)	(0.02)
Observations	63	63	63	27	27	27
R-squared	0.816	0.912	0.941	0.986	0.991	0.991

Note: Robust standard errors in parentheses;

*** p<0.01,

* p<0.10

To investigate the sensitivity of the parameter estimates a range of robustness checks were done. The time trend was replaced by log population. This does not affect the main findings. Other sensitivity analyses are the following. The performance indicators were replaced by performance indicators from the previous season. The parameters were estimated in one step rather than in two stages. Points achieved were included separately before 1995/96 and from 1995/96 onward, since from 1995/96 onward a win generated three points rather than two points as was the case up to that season. A quadratic time trend was introduced. The ranking indicators was specified separately by league (Eredivisie, first division, second division). Club-specific time trends were introduced. In none of these sensitivity analyses the main findings were affected.

### 4.3 An exploratory analysis of international trends

The main conclusion from the empirical analysis in the previous section is that the dip in stadium attendance in the Netherlands is due to a combination of socioeconomic developments as represented by unemployment rates and international spillovers as represented by stadium attendance in the Premier League. This conclusion is based on an analysis of stadium attendance of a balanced panel of 30 football clubs. One of the issues to consider is whether a balanced panel generates a bias in the parameter estimates compared to an unbalanced panel in which all professional football clubs would have been involved. After all, it is possible that surviving clubs have a more stable attendance while clubs that disappeared were affected more by cyclical fluctuations or performance indicators.

To investigate the sensitivity of the main findings an additional analysis is done in which seasonal average in stadium attendance in the Eredivisie is the dependent variable. This exploratory analysis is also done for the top leagues of England, Germany, France and Belgium. Since Italy is an outlier, i.e. it has a development in stadium attendance that is very different from the other countries it is not included in the analysis. The developments of the dependent variables are shown in Figs 1 and 2. The exploratory analysis is done in three steps. First, stadium attendance is related to unemployment rates and a time trend. Second, the (log of) stadium attendance in the English Premier League is included as a right-hand side variable. Third, the (log of) average stadium attendance in the other four countries is included as a right-hand side variable. Because of the potential international spill-over effects the equations are estimated over the time period for which there is information for all countries, i.e. from 1963/64 to 2018/19.

Panel a of Table 6 shows the parameter estimates of unemployment rate and a time trend ignoring potential international spillover effects. Both are significantly different from zero for every country. The negative effect on unemployment on stadium attendance is the strongest in the Netherlands and England and the weakest in Germany and France. The calendar time trend is upward sloping and significant in every country, the smallest in magnitude for Belgium.

**Table 6:** Parameter estimates stadium attendance; average annual attendance top leagues; 1963/64-2018/19.

	Netherlands	England	Germany	France	Belgium
	(1)	(2)	(3)	(4)	(5)
a. No international spillovers					
Log Urate	-0.44***	-0.31***	-0.12***	-0.12***	-0.21***
	(0.04)	(0.03)	(0.03)	(0.03)	(0.03)
Time/10	0.20***	0.12***	0.20***	0.25***	0.07***
	(0.02)	(0.01)	(0.01)	(0.01)	(0.01)
R-squared	0.817	0.794	0.769	0.884	0.510
b. English Premier League					
Log Urate	-0.17***		-0.00	0.08	-0.09
	(0.05)		(0.03)	(0.08)	(0.06)
Time/10	0.09***		0.11***	0.16***	0.01
	(0.02)		(0.01)	(0.04)	(0.02)
English PL	0.96***		0.64***	0.51***	0.42***
	(0.125)		(0.09)	(0.17)	(0.13)
R-squared	0.921		0.855	0.903	0.587
c. Other four leagues					
Log Urate	-0.13***	-0.14***	0.00	0.16**	-0.13**
	(0.04)	(0.03)	(0.03)	(0.08)	(0.05)
Time/10	-0.01	-0.00	0.08***	0.09**	0.01
	(0.03)	(0.02)	(0.02)	(0.04)	(0.03)
Average other leagues	1.38***	0.67***	0.75***	0.71***	0.32**
	(0.15)	(0.12)	(0.09)	(0.17)	(0.13)
R-squared	0.940	0.876	0.872	0.903	0.550

Note: The variable “International spillovers” is defined as the log of the average stadium attendance in the other four leagues. Robust standard errors in parentheses;

*** p<0.01,

** p<0.05

Panel b of Table 6 shows parameter estimates if (log of) attendance in the Premier League is included as additional right-hand side variable. For each of the countries there is a positive and significant parameter estimate of the Premier League attendance. The time trends are all smaller because some of the upward trend is now picked up by the Premier League variable. The effect on unemployment rate of introducing the Premier League attendance differs a lot between countries. For the Netherlands the unemployment effect is still negative and significantly different from zero but the magnitude is much smaller than in panel a. For Germany and Belgium the sign of the unemployment rate variable is still negative but the parameter is now not significantly different from zero. For France the parameter estimate of the unemployment rate is positive though insignificantly different from zero.

Panel c of Table 6 shows parameter estimates if instead of attendance in the Premier League the average attendance in the other four leagues is introduced as a right-hand side variable. For the Netherlands, England and Belgium the effect of unemployment is negative and significant, for Germany there is no unemployment effect while for France the unemployment now has a significant positive effect. The calendar time effects disappear for the Netherlands, England and Belgium but are still positive and significant for Germany and France. For every country, the parameter estimates for (log) average attendance in the other four countries are positive and significantly different from zero. Comparing the parameter estimates of the unemployment rate in panels a and c, it is clear that there is a high correlation between the unemployment rates and the international spillovers. This is due to the high correlation in unemployment rates across the five countries. Therefore, in Netherlands, England and Belgium the parameter estimates of the unemployment rate are reduced but still significantly different from zero while in Germany the effect disappears and in France the effect becomes positive. As additional explanatory variable for German stadium attendance a dummy variable was used for post German unification with a value of 0 up to 1990 and a value of 1 from 1990 onward. The related parameter estimate is positive and significantly different from zero but the other parameter estimates are hardly affected.

**Fig 5:**
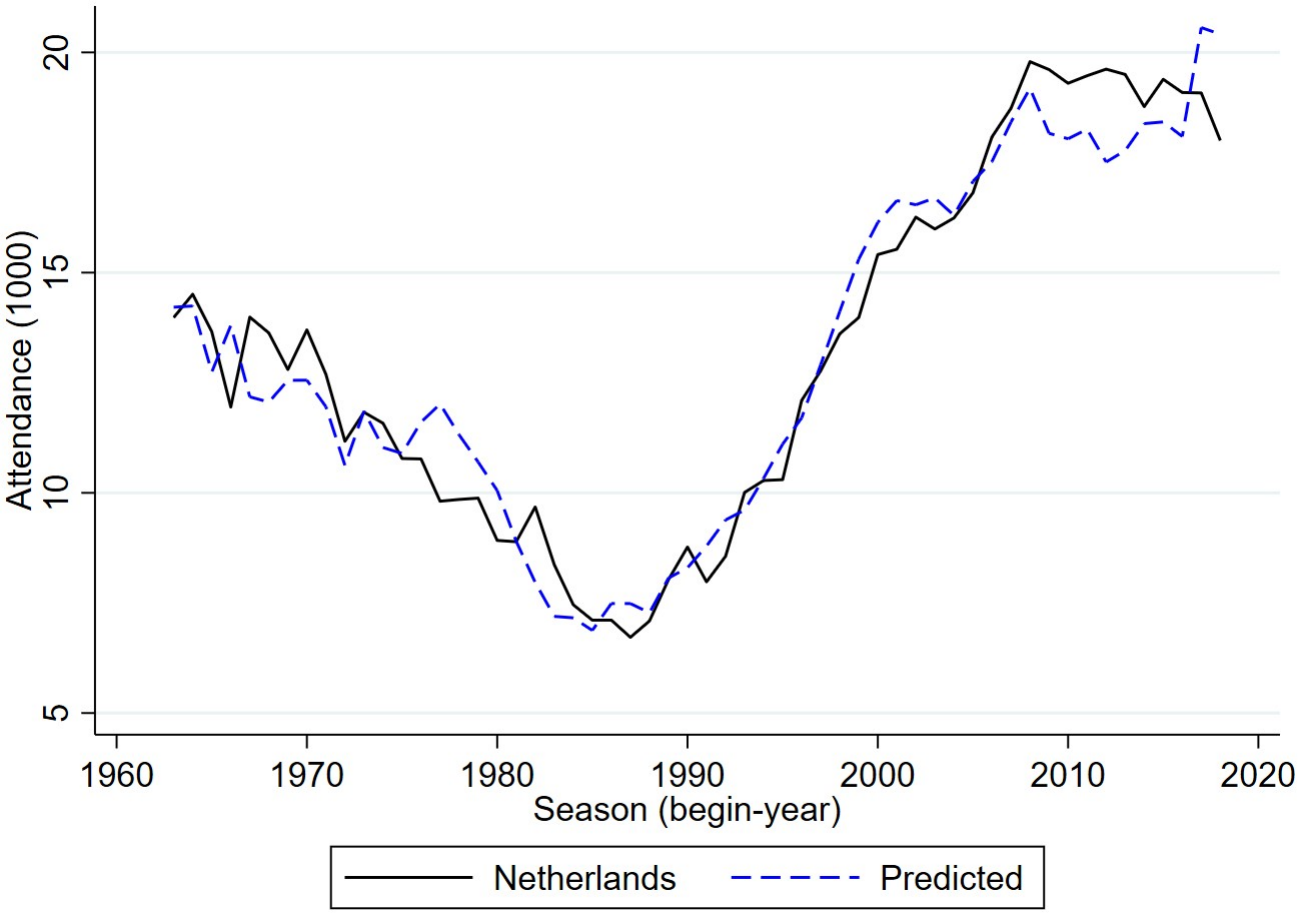
Stadium attendance professional football Netherlands; actual and predicted attendance; 1963/64-2018/19 (1000).

**Fig 6:**
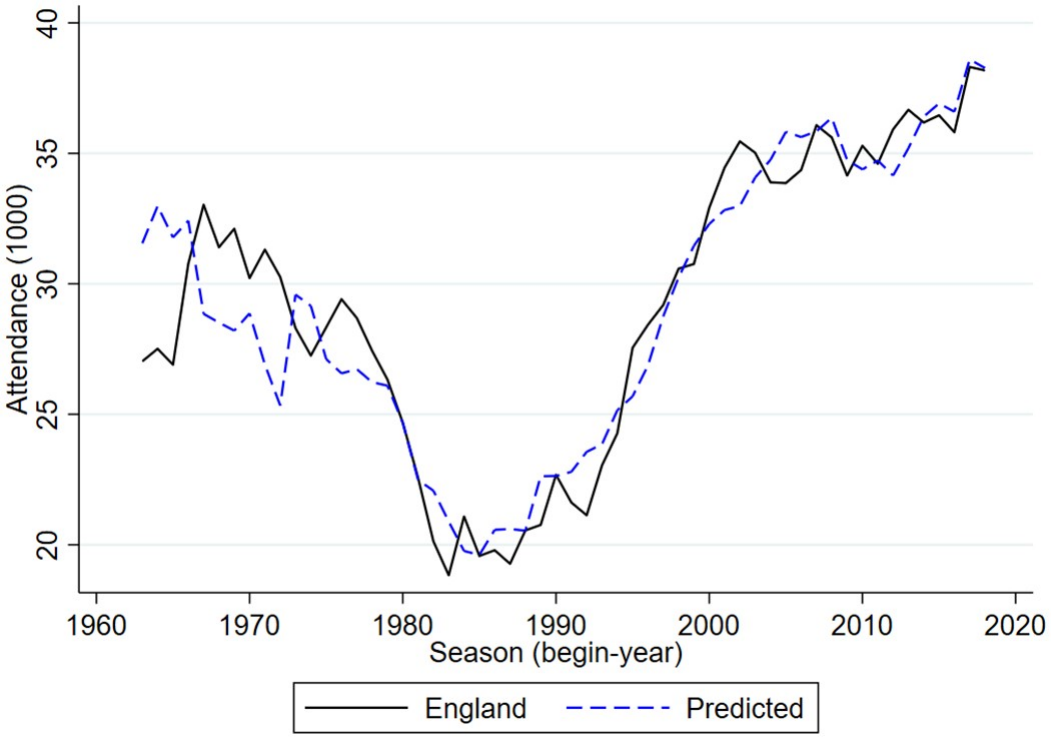
Stadium attendance professional football England; actual and predicted attendance; 1963/64-2018/19 (1000).

**Fig 7:**
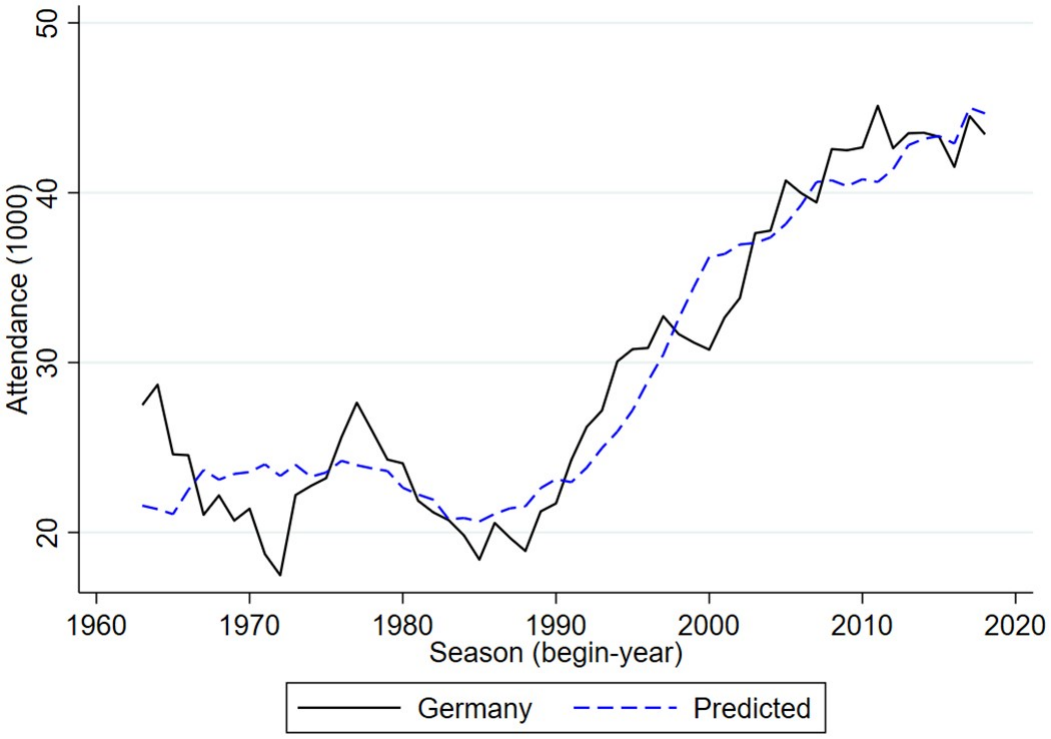
Stadium attendance professional football Germany; actual and predicted attendance; 1963/64-2018/19 (1000).

**Fig 8:**
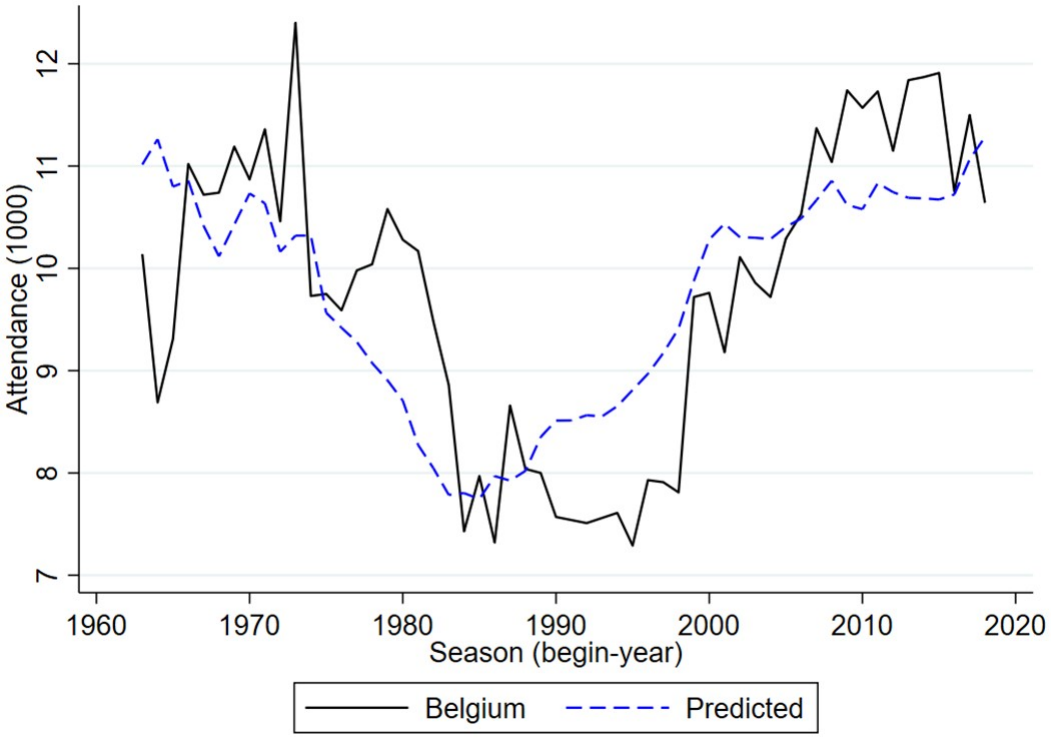
Stadium attendance professional football Belgium; actual and predicted attendance; 1963/64-2018/19 (1000).

**Fig 9:**
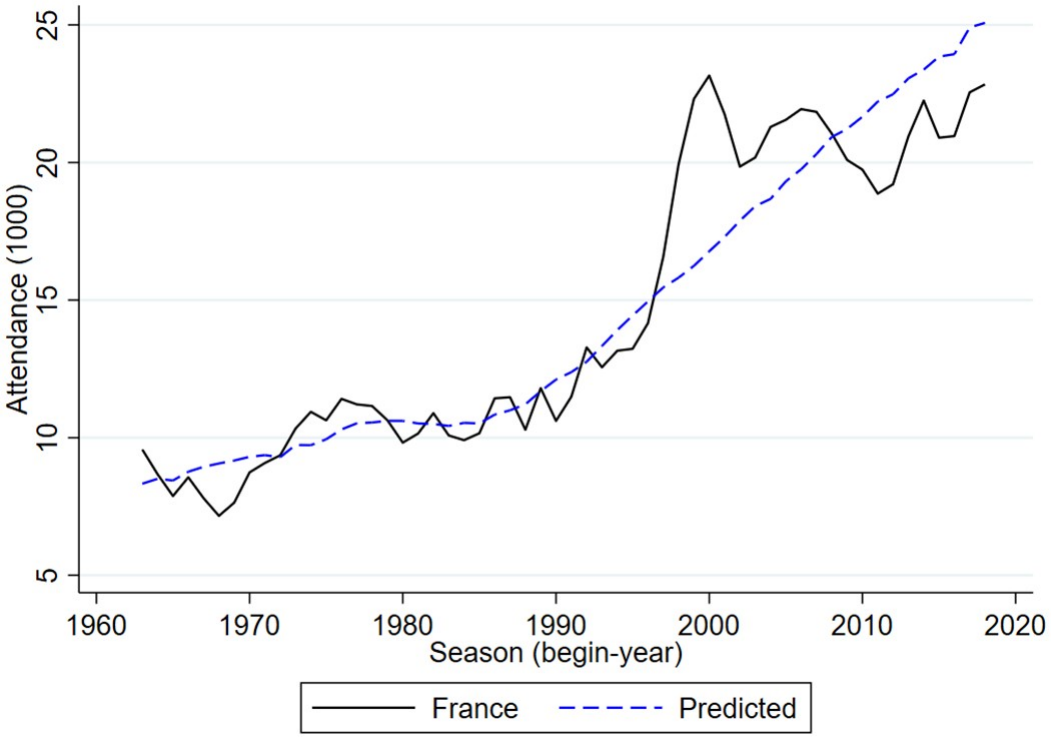
Stadium attendance professional football; actual and predicted attendance France; 1963/64-2018/19 (1000).

Figs 5–9 compare the actual developments in stadium attendance in the four countries with the predicted developments according to the estimates in panel c of Table 1 (except for France where the parameter estimates of panel b are used). Clearly, for the Netherlands and England the predictions are quite accurate, for Germany they are not too bad, for Belgium they are worse and for France they are not very accurate. With a simple model in which developments in football stadium attendance are related to national unemployment rates and international developments, general trends in Netherlands, England and Germany can be explained but this is less so in Belgium and France. In these countries specific developments may have affected stadium attendance. To explore the nature of these developments is beyond the scope of the current paper.

## 5 Conclusions

Professional football attracts a lot of attention from media, television viewers and supporters who visit stadiums on a regular basis. It is no exception that tens of thousands of people travel to a stadium to watch their favorite team play for two times 45 minutes. Since many matches can be watched on television as well it is surprising that so many football fans are willing to spend four to six hours of their life just to watch their team play and spend quite a lot of money too. After all, it is not just watching the game but also traveling to and from the stadium that is time consuming. And, it is not just the price of the ticket but also the travel costs that have to be covered. The phenomenon of massive interest in football stadium attendance goes back a long time. Fifty to sixty years ago the situation was not very different. Even in those days with substantially lower incomes, longer travel times as public transport was for many the only possible way to reach the stadium, uncomfortable seats in sometimes appalling weather conditions visiting a stadium was very popular. Looking back at the developments in stadium attendance over the past 50 to 60 years there is a remarkable dip in the 1980s, a phenomenon that the Netherlands shares with England, Germany, France, and Belgium.

The current paper presents an analysis of long-term developments in professional football stadium attendance in the Netherlands for a balanced panel of 30 clubs over 63 seasons. Stadium attendance appears to be influenced by club-specific factors and season-specific determinants. At the level of the club, stadium attendance is affected by stadium capacity, performance of the club and the league in which the club plays. In this analysis the assumption is that the league effects are constant over time, i.e. possible substitution effects between league induced by commercialization causing a shift in attendance from lower leagues to higher leagues are not accounted for.

Over time, unemployment rates seem to have been important but not hooliganism. It should be noted that changes in unemployment rates also represent changing socioeconomic circumstances such as fluctuations in income. The absence of a hooliganism effect may be specific for the Netherlands as in other countries the nature and severity of hooliganism may have been different. In addition to this, there is a strong association between stadium attendance in the Netherlands and the top football leagues in other European countries in particular England. Part of this association may be due to common determinants such as unemployment rates that are strongly correlated across Europe. Nevertheless, there seem to be unobserved factors influencing both football leagues through international common trends in the interest for professional football. The nature of these unobserved international spillover effects is not clear. It could be shared interest in football as a sport. From an exploratory analysis it clear that a simple model with unemployment rates and stadium attendance in other countries also goes a long way in describing developments in football stadium attendance in the Netherlands, England, Belgium and Germany but less so in France.

The future of football stadium attendance is unclear. The current Covid-19 pandemic with its lockdowns is responsible for empty stadiums. Football supports who used to share their joy and excitement as well as sadness and disappointment now have to digest all these emotions alone or with a few friends. So sad. From the analysis presented it is clear that unemployment has a negative effect on stadium attendance. Thus, Covid-19 related growth of unemployment will have a negative effect on future stadium attendance. Nevertheless, as yet, the stadiums are still empty. It is not clear how quickly football lovers are allowed to return to the stadium. It might be gradually, starting with a low occupancy rate to allow for sufficient distance between the spectators. However, even a gradual return to the stadium would bring immense joy to the ones who are allowed to watch their favorite team face to face. Viewing a match on television is no doubt more comfortable, time efficient and cheaper than visiting a stadium. Nevertheless, it is not a real substitute for the live event.

## Supporting information

S1 Appendix (PDF)
