# Supplementary material for: Common international trends in football stadium attendance
Source: PLoS One. 2021 Mar 3;16(3):e0247761. doi: 10.1371/journal.pone.0247761 (PMC7928532; doi:10.1371/journal.pone.0247761)
Supplement: S1 Appendix — (PDF) [file pone.0247761.s001.pdf]

Appendix: Additional information on the data

1. Creating a coherent series of club observations

Over the years, quite a few clubs ceased to be while other clubs merged into a new club with a different name. Sometimes a merger of two clubs got the name of one of the clubs. In order situations, clubs changed their name to emphasize the name of the city of residence or introduced small changes in their name.

1.1 Mergers

For the teams that have undergone name changes that were not the result of a merger of two or more teams, the most recent name was used. If a merger occurred at any point between 1956/57 and 2018/19, one team is considered to be the 'predecessor' of the post-fusion club. If the 'predecessor' played in all seasons and at least once in the Eredivisie (either as the original club or as the merger club), their team name was replaced by the name of the post-merger club in all seasons. The other teams that contributed to the mergers are not considered in this analysis. The name changes resulting from football club mergers are listed below (first club is considered to be the predecessor of the merger):

1962: Rapid JC, Roda Sport: Roda JC Kerkrade
1963: Stormvogels, VSV: Telstar
1965: SC Enschede, Enschedese Boys: FC Twente ('65)
1967: Alkmaar '54, FC Zaanstreek: AZ Alkmaar
1967: FC Den Bosch, Wilhelmina: FC Den Bosch ('67)
1968: Fortuna '54, RKSV Sittardia: Fortuna Sittard
1969: PEC, Zwolsche Boys: PEC Zwolle
1970: DOS, USV Elinkwijk, Velox: FC Utrecht
1971: ADO, Holland Sport: ADO Den Haag
1991: Dordrecht '90, SVV Schiedam: FC Dordrecht

1.2 Variety of names

The names of most clubs have changed over time. Here is an overview:

1. ADO Den Haag, ADO, FC Den Haag
2. Ajax, AFC Ajax, AFC Ajax Amsterdam
3. AZ Alkmaar, (VV) Alkmaar '54, AZ '67 (Alkmaar), AZ
4. De Graafschap
5. FC Den Bosch, BVV (Den Bosch), FC Den Bosch '67
6. FC Dordrecht, DFC, DS'79, Drechtsteden'79, Dordrecht'90, SVV/Dordrecht'90

7. FC Eindhoven, (SBV/SC) Eindhoven, EVV
8. FC Groningen, Groninger VAV, GVAV (Groningen)
9. FC Twente, SC Enschede, Sportclub Enschede
10. FC Utrecht, VV DOS, DOS Utrecht
11. FC Volendam, Volendam
12. Feyenoord, Feijenoord
13. Fortuna Sittard, Fortuna '54, Fortuna SC, FSC Geleen
14. Go Ahead Eagles, Go Ahead, Go Ahead Eagles Deventer
15. Helmond Sport, Helmondia '55
16. Heracles Almelo, SC Heracles '74
17. MVV Maastricht, MVV
18. NAC Breda, NAC
19. NEC Nijmegen, N.E.C.
20. PEC Zwolle, PEC Zwolle '82, FC Zwolle, PEC
21. PSV
22. Roda JC Kerkrade, Rapid JC Heerlen, Roda JC
23. SBV Excelsior, Excelsior
24. SBV Vitesse, Vitesse
25. SC Cambuur, (Cambuur) Leeuwarden,
26. sc Heerenveen, Heerenveen, SC Heerenveen
27. Sparta Rotterdam
28. Telstar, SC Telstar, IJVV Stormvogels
29. VVV-Venlo,(FC) VVV
30. Willem II, Willem II (1896) Tilburg

2. Variables used in the analysis

2.1 Club-specific variables

- Attendance: Seasonal average match attendance
- Capacity: Highest match attendance during the season
- First Division: Dummy variable for a first division club
- Second Division: Dummy variable for a second division club
- Points/100: Number of points end of season divided by 100; 3 points for a win, 1 point for a draw, 0 points for a loss
- Ranking/100: End of season rank divided by 100
- Goal difference/100: End of season difference goals scored and goals conceded

2.2 Season/year-specific variables

- Unemployment rate: Unemployed as a percentage of the labor force
- Premier League: Average match attendance in the Premier League
- Arrests: Number of arrests because of football hooliganism
- Cinema: Cinema visits (mln)

2.3 Sources

- Attendance: www.european-football-statistics.co.uk/attn.htm
- Performance:
  - Eredivisie: www.voetbal.com/wedstrijd/ned-eredivisie/
  - First Division: 1956/57 - 1995/96 nl.wikipedia.org/wiki/Eerste_divisie_(voetbal_Nederland); 1996/97 - 2018/19 www.voetbal.com/wedstrijd/ned-eerste-divisie/
  - Second Division: nl.wikipedia.org/wiki/Tweede_divisie_(voetbal_Nederland)
- Unemployment rate: Statistics Netherlands
- Arrests football hooligans: Centraal Informatiepunt Voetbalvandalisme
- Cinema: boekman.nl/actualiteit/cijfers-in-context/nieuwe-dataset-bioscoopgeschiedenis-in-cijfers
- Unemployment rates international: OECD Labor Force Statistics
